# Impact of COVID-19 Pandemic on Healthcare-Associated Infections: A Systematic Review and Meta-Analysis

**DOI:** 10.3390/antibiotics12111600

**Published:** 2023-11-07

**Authors:** Usman Abubakar, Ahmed Awaisu, Amer Hayat Khan, Khurshid Alam

**Affiliations:** 1 Department of Clinical Pharmacy and Practice, College of Pharmacy, QU Health, Qatar University, Doha P.O. Box 2713, Qatar; 2 Discipline of Clinical Pharmacy, School of Pharmaceutical Sciences, Universiti Sains Malaysia, George Town 11800, Malaysia

**Keywords:** healthcare-associated infections, COVID-19 pandemic, central line-associated bloodstream infections, catheter-associated urinary tract infection, surgical site infection, *Clostridium difficile* infection, hospital-acquired pneumonia, ventilator-associated pneumonia

## Abstract

This study investigated how the Coronavirus Disease 2019 (COVID-19) pandemic has affected the rate of healthcare-associated infections (HAIs). PubMed, Scopus and Google Scholar were searched to identify potentially eligible studies published from December 2019 to September 2022. A random effect model was used to determine the changes in the rate of HAIs during the pandemic. Thirty-seven studies, mostly from the United States (*n* = 13), were included. Fifteen studies described how the pandemic affected the rate of CLABSIs and CAUTIs, and eight of them showed a significant increase in CLABSIs. The risk of CLABSIs and CDIs was 27% (pooled odds ratio [OR]: 0.73; confidence interval [CI]: 0.61–0.89; *p* < 0.001) and 20% (pooled OR: 1.20; CI: 1.10–1.31; *p* < 0.001) higher during the pandemic compared to before the COVID-19 pandemic period, respectively. However, the overall risk of HAIs was unaffected by the pandemic (pooled OR: 1.00; 95 CI: 0.80–1.24; *p* = 0.990). Furthermore, there were no significant changes in the risk of CAUTIs (pooled OR: 1.01; 95 CI: 0.88–1.16; *p* = 0.890), and SSIs (pooled OR: 1.27; CI: 0.91–1.76; *p* = 0.16) between the two periods. The COVID-19 pandemic had no effect on the overall risk of HAIs among hospitalized patients, but an increased risk of CLABSIs and CDI were observed during the pandemic. Therefore, more stringent infection control and prevention measures and prudent interventions to promote the rational use of antibiotics are warranted across all healthcare facilities to reduce the burden of HAIs.

## 1. Introduction

One of the major patient safety concerns during hospitalization is the occurrence of healthcare-associated infections (HAIs). This is because HAIs cause an increase in morbidity, mortality, and healthcare-associated cost [1]. There are variations in the rate of HAIs between countries, with 4% in the United States (US) [2], 6.5% in Europe [3], 9.0% in Asia [4], and approximately 16% in developing countries [5]. Africa has a two-fold higher rate of HAIs as compared to the developed countries [6,7]. HAIs are potentially preventable through compliance with infection control and prevention recommendations [1]. Hand hygiene is the mainstay for the prevention of HAIs and this is beneficial in reducing the transmission of multidrug-resistant organisms [8]. Infection control and prevention programs were disrupted during the COVID-19 pandemic, and this has a potential impact on the incidence of HAIs and transmission of multidrug-resistant organisms. The rate of multidrug-resistant Gram-negative and Gram-positive pathogens has increased during the COVID-19 pandemic [9]. Prior to the COVID-19 pandemic, compliance with recommendations from guidelines on hand hygiene was poor among healthcare workers [10]. However, improved hand hygiene and environmental hygiene was reported during the COVID-19 pandemic [11], and this could potentially reduce the rate of HAIs and transmission of multidrug-resistant organisms.

Conversely, hospital resources, including infection prevention and control resources, were diverted to the management of the COVID-19 pandemic, and this could potentially affect the compliance with infection control and prevention recommendations leading to an increase in the rate of HAIs [12]. The diversion of hospital resources may potentially nullify the benefits of improved hand hygiene on the rate of HAIs during the COVID-19 pandemic [13]. In addition, there was a decline in hospital visits and overcrowding due to the enforcement of movement restrictions during the pandemic, and this could potentially benefit infection prevention and control programs [14,15]. Furthermore, the transmission of hospital-acquired respiratory pathogens was reduced due to the increase in the use of face masks by healthcare workers and patients [15]. Currently, the effect of the COVID-19 pandemic on the rate of HAIs is a subject of debate. While some believe that COVID-19 mitigation strategies could potentially reduce the rate of HAIs [13,16], others have argued that the diversion of hospital resources during the pandemic could potentially increase the rate of HAIs [13]. This study aimed to synthesize the effect of the COVID-19 pandemic on the overall risk of HAIs, and determine the effect of the pandemic on the risk of individual types of HAIs, including central line-associated bloodstream infections (CLABSI), catheter-associated urinary tract infections (CAUTI), *Clostridium difficile* infection (CDI), surgical site infections (SSI), and ventilator-associated pneumonia/hospital-acquired pneumonia (VAP/HAP).

## 2. Materials and Methods

### 2.1. Study Design

The Preferred Reporting Items for Systematic Review and Meta-Analysis (PRISMA) statements 2020 was used to conduct and report this systematic review [17]. The study protocol was registered with PROPSPERO (reference ID: CRD42023463262).

### 2.2. Eligibility Criteria

#### 2.2.1. Inclusion Criteria

A study was included if it fulfilled the following predefined criteria:

- Compared the rate of HAIs between the periods before the pandemic and during the pandemic using interrupted time series or before and after study design;
- Published between December 2019 and September 2022;
- Published in English language;
- Available as free full-text article.

#### 2.2.2. Exclusion Criteria

A study was excluded if it fulfilled any of the following criteria:

- Described nosocomial transmission of COVID-19 infections;
- Preprints, correspondence, commentary, and letters to the editor;
- Qualitative studies.

### 2.3. Information Sources

PubMed and Scopus databases were searched by two reviewers to find potentially eligible studies. Supplementary search of Google Scholar was conducted to identify eligible studies. The reference list of the selected studies was manually examined to find additional studies.

### 2.4. Search Strategy

The relevant keywords for HAIs and the COVID-19 pandemic were combined using Boolean indicators (AND/OR). The following keywords were used for the search: impact OR effect OR change AND “hospital-acquired infection^*^” OR “healthcare-associated infection^*^” OR “nosocomial infection^*^” AND “SARS-CoV-2” OR “COVID-19” OR “coronavirus disease 2019” OR “severe acute respiratory syndrome coronavirus 2” OR “coronavirus infection” OR “coronavirus pandemic” OR “COVID-19 pandemic”.

### 2.5. Selection Process

The results of the searches from all the databases were combined in one folder and duplicate studies were removed. The titles and abstracts of the studies were initially assessed and irrelevant studies were excluded. The full-text articles of the remaining studies were assessed based on the inclusion and exclusion criteria for selection and data extraction.

### 2.6. Data Extraction Process

The included studies were reviewed for data extraction using a predefined data collection form. Data extraction was performed by an independent reviewer (UA) and the extracted data were checked by a second reviewer for accuracy. All disagreements were resolved by the reviewers through dialogue.

### 2.7. Data Items

Data items extracted from the included studies include: name of author and year of publication, study location, study setting, the study design, study period, sample size, hospital units involved, rate of HAIs before and during the COVID-19 pandemic, type of HAIs, and the *p*-value. In addition, the frequency of HAI, the number of patients, the total patient days and total device days (for urinary catheter and central catheter) for both periods were extracted.

### 2.8. Study Risk of Bias Assessment

Methodological quality of the included studies was assessed by two independent reviewers (AHY and KA) using the Newcastle–Ottawa scale (NOS) [18]. NOS consists of three sections including: selection, comparability, and outcomes. The reviewers resolved any discrepancies through dialogue.

### 2.9. Outcome Assessment and Effect Measures

The primary outcome was the effect of the COVID-19 pandemic on the overall risk of HAIs, and this was determined by comparing the overall rate of HAIs before versus during the COVID-19 pandemic. The Centers for Disease Control and Prevention (CDC) [19] and the European Centres for Disease Prevention and Control (ECDC) guidelines were used to define HAIs [20]. The secondary outcomes assessed include the risk of CLABSI, CAUTI, CDI, SSI, and VAP/HAP presented as odds ratio with 95% confidence interval. These infections are referred by CDC as types of HAIs.

### 2.10. Data Synthesis

Both qualitative and quantitative synthesis was used. Review Manager (RevMan) [Computer program], version 5.4. The Cochrane Collaboration, 2020 was used for the quantitative synthesis. The pooled estimate was determined using random-effects meta-analysis, and the findings were presented using forest plots. Higgins I^2^ statistic was employed to assess the level of heterogeneity using the following criteria; <40% = low heterogeneity, 30–60% = moderate heterogeneity, 50–90% = substantial heterogeneity, and 75–100% considerable heterogeneity [21]. The overall rate of HAIs was evaluated as the number of patients with HAI as a proportion of all hospitalized patients. The overall risk of HAIs was estimated by comparing the overall rate of HAIs before versus the rate during the COVID-19 pandemic. Furthermore, the risk for the different types of HAIs (CLABSI, CAUTI, SSI, CDI, and HAP/VAP) was estimated by comparing the rate of HAIs (number of events divided by the total patient days or total-device days) between the period before and the period during the COVID-19 pandemic. For each type of HAI, data were meta-analyzed when at least two studies reported that particular HAI.

## 3. Results

### 3.1. Study Selection

**Figure 1:**
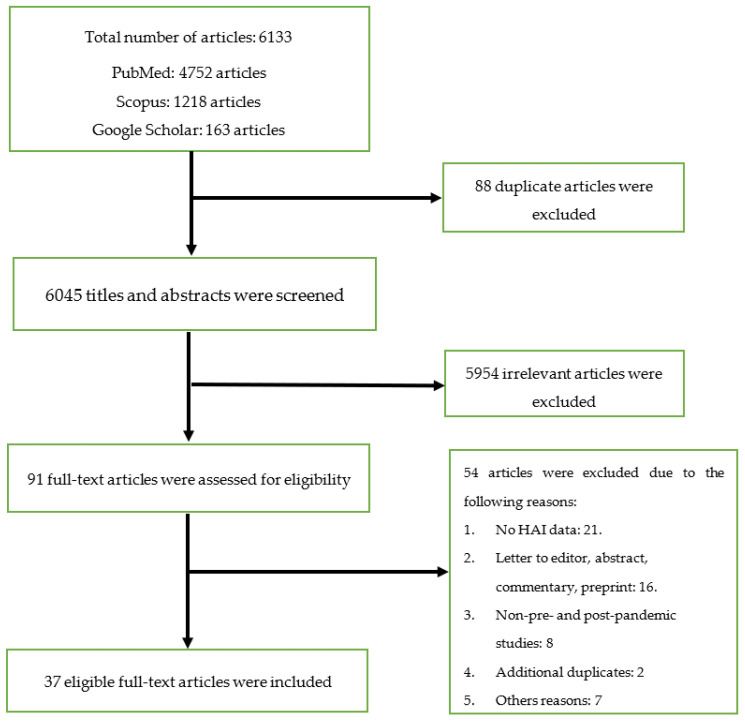
Flow chart for the screening and selection processes.

The database searches produced 6133 articles, out of which 88 duplicates were removed. The title and abstract of the de-duplicated articles was screened and 5954 irrelevant articles were excluded. The remaining 91 full-text articles were evaluated for inclusion, and 37 articles that fulfilled the criteria were eventually selected. Figure 1 illustrates the PRISMA flow diagram for the screening and selection process.

### 3.2. Study Characteristics

North America (*n* = 14; 37.8%), Europe (*n* = 11; 29.7%), and Asia (*n* = 5; 13.5%) had the highest number of studies. The US had the highest number of studies (*n* = 13; 35.1%) followed by Italy (*n* = 4; 10.8%), and Spain (*n* = 3; 8.1%). Most of the studies (*n* = 27; 72.9%) included hospital-wide data, while four studies (10.8%) involved data from intensive care units (ICUs) only. Furthermore, the majority of the studies (*n* = 26; 70.3%) included multiple study centers. Six studies compared the overall prevalence of HAIs between the period before the COVID-19 pandemic and during the pandemic [22,23,24,25,26,27]. CLABSIs (*n* = 15; 40.5%) [28,29,30,31,32,33,34,35,36,37,38,39,40,41,42], CAUTIs (*n* = 15; 40.5%) [22,28,30,31,32,33,34,35,36,39,40,41,42,43,44], and CDI (*n* = 14; 37.8%) [28,30,31,32,34,36,40,43,45,46,47,48,49,50] were the most reported HAIs in the selected studies. Table 1 presents the characteristics of the studies included in this review.

### 3.3. Quality Assessment of the Studies

Most of the included studies had a truly or somewhat representative target population. In addition, the sample size for most of the studies was satisfactory and justified. The quality score for the included studies ranged from 6 to 7, with 33 studies (89.2%) scoring 7 points. Overall, the methodological quality was good in the majority of the studies (89.2%), although, four studies were found to have a fair methodological quality. Table 2 illustrates the quality assessment results of the included studies.

### 3.4. Qualitative Summary of Results

#### 3.4.1. The Effect of COVID-19 Pandemic on Overall Rate of Healthcare-Associated Infections (HAIs)

Six studies reported the overall effect of the pandemic on the HAIs [22,23,24,25,26,27]. Four of them showed a 7.6% to 66.4% increase in the overall rate of HAIs during the pandemic [22,24,25,27]. However, two studies reported an overall reduction in HAIs during the pandemic [23,26].

#### 3.4.2. The Effect of COVID-19 Pandemic on Central Line-Associated Bloodstream Infections (CLABSIs)

The effect of the pandemic on CLABSIs was described in 15 studies [28,29,30,31,32,33,34,35,36,37,38,39,40,41,42]. The majority of the studies (*n* = 11, 73.3%) showed an increase in the rate of during the COVID-19 pandemic, and the increase ranged from 27.9% to 192.6% [28,29,31,32,33,34,37,38,39,40,41]. Of these studies, eight reported a statistically significant increase in CLABSIs during the pandemic [28,29,31,32,33,37,38,39]. Four studies reported a decrease in CLABSIs during the pandemic [30,35,36,42], but only one was statistically significant [30].

#### 3.4.3. The Effect of COVID-19 Pandemic on Catheter-Associated Urinary Tract Infections (CAUTIs)

Similarly, 15 studies reported the impact of the COVID-19 pandemic on CAUTIs [22,28,30,31,32,33,34,35,36,39,40,41,42,43,44]. Seven studies demonstrated a 10.5% to 46.8% decrease in CAUTIs during the pandemic [28,33,35,36,42,43,44], while three studies reported a 20.5% to 74.7% increase in CAUTIs during the pandemic [31,34,41]. Two studies showed that there was no change in the rate of CAUTIs during the pandemic [30,32].

#### 3.4.4. The Effect of COVID-19 Pandemic on Healthcare-Associated Clostridium Difficile Infection (CDI)

Of the 14 studies that reported this outcome, 12 studies (85.7%) showed a 4.9% to 88.2% decrease in the rate of healthcare-associated CDI during the pandemic [28,30,32,34,36,43,45,46,47,48,49,50]. However, only four of them demonstrated a significant reduction in CDI during the pandemic [32,46,49,50]. One study reported a non-statistically significant increase in the rate of CDI during the pandemic [31].

#### 3.4.5. The Effect of COVID-19 Pandemic on Surgical Site Infections (SSIs)

Overall, the impact of the COVID-19 pandemic on SSIs was reported in seven studies [23,25,27,40,43,54,56]. Four of them showed a 14.2% to 60.7% decrease in SSIs during the pandemic [23,43,54,56], and only two studies showed a significant reduction in SSIs [23,56]. Conversely, Chen et al. reported an increase in SSIs from 11.8% to 14.8% during the pandemic (*p* = 0.084) [25].

#### 3.4.6. The Effect of COVID-19 Pandemic on Ventilator-Associated Pneumonia

Four studies reported the effect of the pandemic on the rate of VAP [35,37,42,43], with two of them showing a significant reduction in VAP during the COVID-19 pandemic [35,42]. Geffer et al. found that the incidence of ventilator-associated lower respiratory tract infections declined from 2.95 before COVID-19 outbreak to 2.02 after COVID-19 outbreak (*p* < 0.001) [35].

### 3.5. Quantitative Summary of Results

#### 3.5.1. Meta-Analysis for the Effect of COVID-19 Pandemic on Overall HAIs

**Figure 2:**
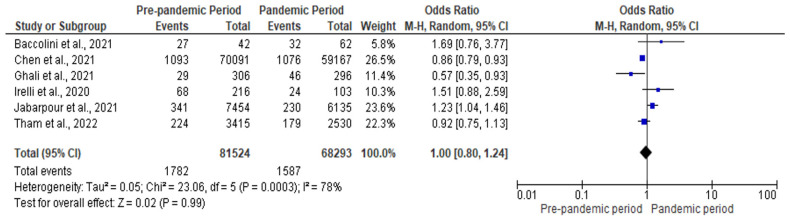
Forest plot for the overall effect of COVID-19 pandemic on HAIs [22,23,24,25,26,27].

All the studies that reported the overall effect of the COVID-19 pandemic on HAIs was included in the meta-analysis. Figure 2 illustrates the forest plot for the effect of the pandemic on the overall risk of HAIs. The pooled estimate showed that the overall risk of HAIs in the pandemic period was similar to the pre-pandemic period (pooled odds ratio [OR]: 1.00; 95 CI: 0.80–1.24; *p* = 0.990). Nevertheless, the level of heterogeneity was high (I^2^ = 78%).

#### 3.5.2. Meta-Analysis for the Effect of COVID-19 Pandemic on CLABSI

**Figure 3:**
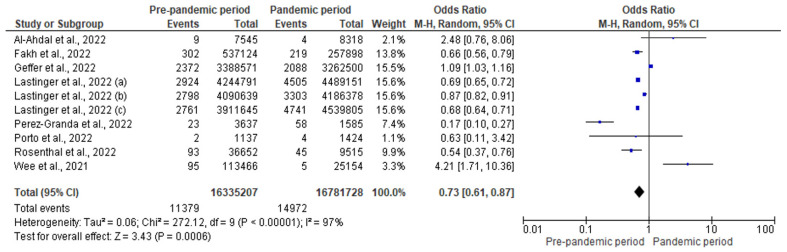
Forest plot for the effect of COVID-19 pandemic on CLABSI [29,30,33,35,37,39,40,42].

A forest plot (Figure 3) revealed that the risk of CLABSI was lower in the pre-pandemic period compared to the pandemic period (pooled OR: 0.73; 95% CI: 0.61–0.89). In other words, the risk of CLABSI was 27% lower in the pre-pandemic period (*p* < 0.001). However, there was a considerable degree of heterogeneity in this analysis (I^2^ = 97%).

#### 3.5.3. Meta-Analysis for the Effect of COVID-19 Pandemic on CDI

**Figure 4:**
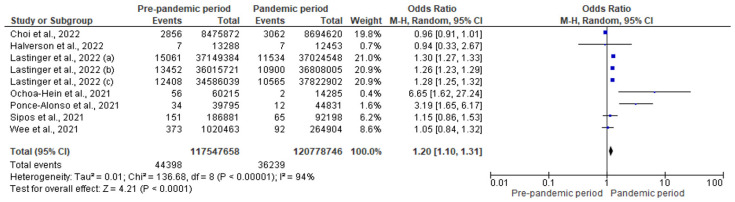
Forest plot for the impact of COVID-19 pandemic on CDI [30,31,40,46,47,49,50].

Figure 4 presents the forest plot for the effect of the COVID-19 pandemic on CDI. In the pre-pandemic period, 44,398 CDIs were reported in 117,547,658 patient days compared to 36,239 CDIs in 120,778,746 patient days observed during the pandemic. This corresponds to a significant 20% increase in the risk of CDI during the pandemic (pooled OR: 1.20; 95% CI: 1.10–1.31; *p* < 0.001).

#### 3.5.4. Meta-Analysis for the Effect of COVID-19 Pandemic on CAUTI

**Figure 5:**
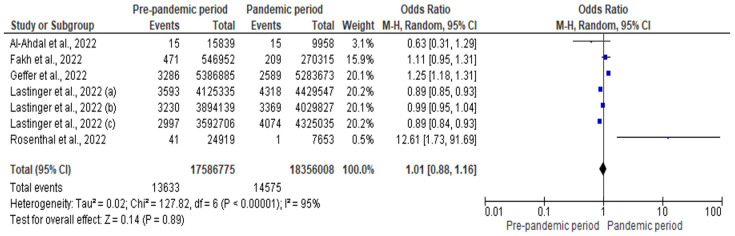
Forest plot for the impact of COVID-19 pandemic on CAUTI [33,35,39,40,42].

The number of CAUTIs was 13,633 and 14,575 during the pre-pandemic and pandemic period, respectively. There were 17,586,775 urinary catheter days in the pre-pandemic period and 18,356,008 urinary catheter days in the pandemic period. Figure 5 shows that there was a non-significant increase in the risk of CAUTI during the pandemic (pooled OR: 1.01; 95% CI: 0.88–1.16; *p* = 0.890; with a high degree of heterogeneity [I^2^ = 95%]).

#### 3.5.5. Meta-Analysis of the Impact of COVID-19 Pandemic on SSI

**Figure 6:**
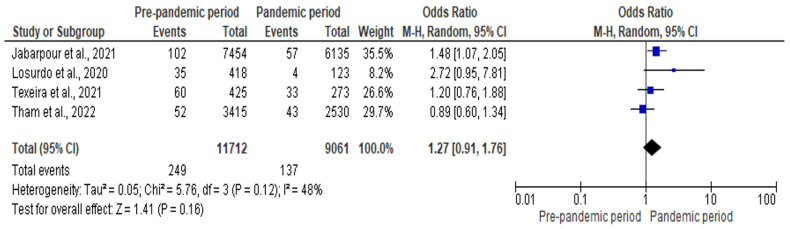
Forest plot for the impact of COVID-19 pandemic on SSI [23,27,54,56].

Four studies involving 11,712 and 9061 patients in the pre-pandemic and pandemic period, respectively, were included in the meta-analysis. The risk of SSI was 27% higher during the pandemic period compared to the pre-pandemic period (OR: 1.27; CI: 0.91–1.76; *p* = 0.16). There was a moderate degree of heterogeneity in this analysis (I^2^ = 48%). Figure 6 represents the forest plot for the meta-analysis of the impact of the COVID-19 pandemic on SSI.

## 4. Discussion

This review examined the effect of the COVID-19 pandemic on the rate of HAIs, and included studies from different continents across the world. The majority of the studies were from North America and Europe with a few studies coming from Africa, Asia, South America, and Oceania. There was no difference in the overall risk of HAIs between the two periods. Conversely, patients hospitalized before the COVID-19 pandemic had a lower risk of CLABSI compared to those in the COVID-19 pandemic period. Similarly, there was a significant 20% increase in the risk of CDI during the COVID-19 pandemic. There was no significant increase in the risk of CAUTI and SSI during the pandemic. Therefore, infection prevention and control programs should be strengthened to reduce the burden of HAIs during and after the pandemic. The available evidence has shown that HAIs, particularly those involving multidrug-resistant organisms, have a high mortality rate [59,60]. There were no variations in the overall risk of HAIs between the two periods, and this implies that COVID-19 mitigation strategies did not affect the overall risk of HAIs. The improvements in hand and environmental hygiene during the COVID-19 pandemic was expected to reduce the incidence of HAIs [16]. However, this potential benefit could be counteracted by the disruption of other infection prevention and control programs such as the surveillance of HAIs, contact precaution and isolation of those colonized with multidrug-resistant pathogens in a separate room [12,13,61]. Therefore, the COVID-19 mitigation strategies that improved hand and environmental hygiene should be sustained, while the infection control measures that were disrupted during the pandemic should be resumed to reduce the incidence of HAIs.

The result also revealed that there was an increase in the risk of CLABSI during the pandemic compared to the period before the pandemic. Generally, hospitalized COVID-19 patients, especially those who are critically ill, have a higher risk of bloodstream infections compared to hospitalized non-COVID-19 patients [62]. This was attributed to the frequent use of a central line, use of immunosuppressive therapy, and reduced compliance with hand hygiene due to increased workload [62,63]. Therefore, improved hand hygiene is recommended to reduce the incidence of CLABSIs [64]. Furthermore, COVID-19 was significantly associated with a higher risk of CDI. CDI has been significantly associated with antibiotic use, the number of prescribed antibiotics, and the duration of antibiotic therapy [65,66,67]. There was a high rate of antibiotic prescription among COVID-19 patients [68,69,70]. The excessive use of antibiotics in COVID-19 patients despite a low rate of secondary infections explains the increase in the risk of CDI during the pandemic [71,72]. Therefore, antimicrobial stewardship is recommended to promote the rational use of antibiotics to reduce the risk of CDI. The effectiveness of antimicrobial stewardship programs in reducing the risk of CDI has been established [73]. In addition, infection control and prevention recommendations should be improved to minimize the horizontal transmission of CDI [74].

The results indicate that there was no significant increase in the risk of CAUTI and SSI during the pandemic. This implies that the infection control recommendations implemented to curb the transmission of COVID-19 did not significantly impact the risk of CAUTI and SSI. In the case of SSI, there are other measures besides infection control recommendations that are used to prevent SSI before, during, and after surgery. Typically, SSIs are preventable through preoperative antimicrobial prophylaxis. Previous studies have shown a low rate of compliance with recommendations for surgical antibiotic prophylaxis before the pandemic [75,76,77]. However, there was an increase in the use of preoperative antimicrobial prophylaxis for genitourinary procedures in the pandemic era compared to the period before the pandemic [78]. In addition to surgical antimicrobial prophylaxis, the duration of surgery, comorbidities such as diabetes and hypertension, tobacco smoking, and the American Society of Anesthesia (ASA) score, are significantly associated with SSIs [79,80,81,82]. These factors could explain the lack of significant improvement in the SSI rate in the pandemic era. Therefore, managing the modifiable risk factors associated with SSI coupled with infection control measures, and surgical antimicrobial prophylaxis is required to reduce the burden of SSI.

The results of this systematic review and meta-analysis should be interpreted with caution in light of some limitations. First, the distribution of the included studies was skewed towards North America and Europe, which accounted for most of the studies and this may affect the generalizability of the findings. However, all the continents were represented in the qualitative and quantitative analyses. Second, there were variations in the definition of HAIs and the classification of HAIs among the included studies, and this is a potential source of assessment and measurement bias. Third, the heterogeneous risk estimates were used by the included studies, where some studies reported the prevalence, while others reported the incidence per 1000 device days or per 1000 patient days. These variations reduced the number of studies included in the meta-analyses, which could potentially affect the findings. However, it is noteworthy that only studies with similar units of measurement were meta-analyzed. In addition, the study period for the included studies was highly variable. While some studies compared the prevalence or incidence in 2019 with 2020, others compared 2019 with 2021. Fourth, the infection prevention and control practices vary from one institution to another and between countries; therefore, the impact of the pandemic on HAIs could be inconsistent. Fifth, most of the studies used a before and after study design, which is associated with a high rate of bias. Sixth, the results for HAP/VAP were not meta-analyzed because the included studies used different units of measurement. Finally, substantial statistical heterogeneity was found in most of the meta-analyses. In spite of the limitations, this study shows evidence of the effect of the COVID-19 pandemic on the risk of HAIs among hospitalized patients.

## 5. Conclusions

The overall risk of HAI was observed to be unaffected by the COVID-19 pandemic. However, the COVID-19 pandemic was significantly associated with a higher risk of CLABSI and CDI. Therefore, more stringent infection prevention and control measures as well as prudent antimicrobial stewardship programs are warranted across all healthcare facilities to reduce the burden of HAIs during such pandemics. Further studies are required from developing countries, especially those in Africa and Asia.

## Figures and Tables

**Table 1:** Characteristics of the studies included in the review.

S/No.	Author and Year	Country and Continent	Study Setting/No of Centers	Study Design	Period of the Study	Number of Participants	Types of HAIs Included	Prevalence/Incidence of HAIs before Pandemic	Prevalence/Incidence of HAIs during Pandemic	*p* Value
1	Irelli et al., 2020 [26]	Italy/Europe	Neurology and stroke unit/single center	Retrospective case–control study	8 March 2020 to 31 May 2020 versus same period in 2019	216 (2019) 103 (2020)	Overall HAI	31.5%	23.3%	0.120
2	Alsuhaibani et al., 2022 [28]	USA/North America	Hospital-wide/single center	NA	2018–2019 versus January–December 2020	NA	CLABSI	0.7–1.4 per 1000 central line days	1.8 per 1000 central line days	0.04
							CAUTI	0.8–1.7 per 1000 catheter days	0.6–1.6 per 1000 catheter days	0.54
							CDI	0.6–1.0 per 10,000 patient days	0.4–0.6 per 10,000 patient days	0.11
3	Sturm et al., 2022 [51]	USA/North America	Hospital-wide/multicenter (69 hospitals)	Before and after	Pre-COVID-19 (1 January 2019 to 28 February 2020), and COVID-19 pandemic period (1 March 2020 to 30 April 2021).	NA	Bloodstream infection	2.78 per 10,000 patient days	3.56 per 10,000 patient days	<0.001
4	Perez-Granda et al., 2022 [29]	Spain/Europe	Hospital-wide/single center	Retrospective before and during the COVID-19 pandemic	March to May 2019 March to May 2020	12,111 versus 10,479 patients.	Catheter-related BSI	1.89 per 1000 admission	5.53 per 1000 admission	<0.001
5	Wee et al., 2021 [30]	Singapore/Asia	Hospital-wide/multicenter	Retrospective before and after	January 2018–January 2020 versus February–August 2020	NA	RVI	9.69 per 10,000 patient days	0.83 per 10,000 patient days	<0.05
							CLABSI	0.83 per 1000 device days	0.20 per 1000 device days	<0.05
							CAUTI	1.8 per 1000 device days	1.8 per 1000 device days	NA
							CDI	3.65 per 10,000 patient days	3.47 per 10,000 patient days	0.66
6	Ochoa-Hein et al., 2021 [47]	Mexico/South America	Hospital-wide/single center	Before–after observational study	January 2019–February 2020 versus April–July 2020	NA	CDI	9.3 per 10,000 patient days	1.4 per 10,000 patient days	NA
7	Polly et al., 2022 [52]	Brazil/South America	Hospital-wide/single center	Retrospective before–after observational study	2017–2019 versus 2020	NA	HCAIs due to MDR bacteria	3.14 per 1000 patient days	3.89 per 1000 patient days	<0.005
8	Halverson et al., 2022 [31]	USA/North America	Hospital-wide/multicenter	Retrospective cohort study	September 2017 to December 2020	NA	CLABSI	0.13 per 1000 patient days	0.24	0.0082
							CAUTI	0.13 per 1000 patient days	0.17	0.052
							CDI	0.52 per 1000 patient days	0.55	0.670
							Overall HAIs	0.80 per 1000 patient days	1.06	0.017
9	Kitt et al., 2022 [53]	USA/North America	Hospital-wide/single center	Retrospective cohort study	July 2017–June 2021	NA	HAVI	0.19 per 1000 patient days	0.06 per 1000 patient days	<0.01
10	Advan et al., 2022 [32]	USA/North America	Hospital-wide/multicenter	Retrospective longitudinal	January 2018–February 2020 versus March 2020–March 2021	NA	CLABSI	0.6 per 1000 catheter days	0.9	0.0023
							CAUTI	0.7 per 1000 catheter days	0.7	0.810
							CDI	3.6 per 10,000 patient days	2.6	<0.001
11	Fakih et al., 2022 [33]	USA/North America	Hospital-wide/multicenter	Retrospective	March 2019–February 2020 versus March–August 2020	NA	CLABSI	0.56 per 1000 line days	0.85	<0.001
							CAUTI	0.86 per 1000 catheter days	0.77	0.190
12	Teixeira et al., 2022 [54]	Portugal/Europe	Urology ward/multicenter	Retrospective observational	April–June 2018 versus April–June 2020	425 patients versus 273 patients	SSI	14.1%	12.1%	0.494
13	Ponce-Alonso et al., 2021 [49]	Spain/Europe	Hospital-wide/single center	Retrospective	Mar–May 2019 versus March–May 2020	39,795 hospital stay (pre) versus 44,831 (pandemic era) hospital stays	CDI	8.54 per 10,000 patient days	2.68 per 10,000 patient days	0.0002
14	Bobbitt et al., 2022 [34]	USA/North America	Hematology and stem cell transplant patients/single center	Retrospective observational	March–July 2019 versus March–July 2020	295 patients versus 259 patients	CDI	2.61 per 1000 patient days	1.58	0.512
							CLABSI	0.44 per 1000 patient days	1.064	0.516
							CAUTI	0.44 per 1000 patient days	0.53	0.899
15	Kong et al., 2021 [36]	USA/North America	Hospital-wide/single center	Retrospective observational	January 2019–February 2020 versus March 2020–June 2020	NA	CDI	0.48 ± 0.12	0.26 ± 0.25	0.200
							CLABSI	1.47 ± 1.63	0.37 ± 0.73	0.210
							CAUTI	1.10 ± 1.18	0.87 ± 0.58	0.720
16	Tham et al., 2022 [27]	Australia	Hospital-wide/single center	Retrospective cohort study	April–June 2019 versus April–June 2020	3415 admission (pre-COVID-19) versus 2530 (COVID-19 era)	Overall HAIs	6.6%	7.1%	NA
							UTI	1.3%	1.6%	NA
							SSI	1.5%	1.7%	NA
							HAP	2.5%	2.3%	NA
							BSI	0.4%	0.4%	NA
							GI	0.4%	0.2%	NA
17	Mohammadi et al., 2022 [55]	Iran/Asia	Hospital-wide/single center	Retrospective study	April–November 2019 versus April–December 2020	16,687 admission (pre pandemic) versus 10,553 admission (pandemic era)	Overall HAIs	4.73%	4.78%	NA
18	Chen et al., 2021 [25]	China/Asia	Hospital-wide/single center	Retrospective before and after	2018–2019 versus 2020	62,625 patients (2018) 70,091 (2019) 59,167 (2020)	Overall HAIs	1.64% (2018) 1.56% (2019)	1.82%	0.001
							LRI	39.5%	39.7%	0.971
							UTI	14.8%	10.5%	0.002
							BSI	11.28%	12.91%	0.079
							SSI	11.83%	14.84%	0.084
							GTI	7.49%	9.62%	0.068
19	Losurdo et al., 2020 [56]	Italy/Europe	Surgery department/single center	Retrospective	2018–2019 versus 2020	418 patients (pre-COVID era) versus 123 (COVID-19 era)	SSI	8.4%	3.3%	0.035
							Superficial SSI	5.3%	0.8%	0.018
							Deep SSI	3.4%	0.0%	0.025
							Organ-space SSI	3.6%	1.6%	0.209
20	Geffer et al., 2022 [35]	Germany/Europe	ICU/multicenter	NA	2019 versus 2020	863,999 patients (2019) and 696,085 patients (2020)	CLABSI	0.7 per 1000 central line days	0.64	0.263
							VALRTI	2.95 per ventilator days	2.02	<0.001
							CAUTI	0.61 per 1000 catheter days	0.49	0.008
21	Porto et al., 2022 [37]	Brazil /South America	ICU/multicenter	NA	April–June 2019 versus April–June 2020	531 (2019) versus 357 (2020)	CLABSI	1.60 per 1000 central line days	2.81	0.002
							VAP	2.99 per 1000 ventilator days	3.65	0.167
22	Samaroo-Campbell et al., 2022 [41]	USA/North America	Hospital-wide/multicenter	Retrospective	15 months before and 15 months after the onset of the pandemic	NA	CLABSI	1.09 ± 0.43 per 1000 catheter days	1.76	NA
							CAUTI	1.03 ± 0.18 per 1000-catheter days	1.80 ± 0.21	0.0003
23	Ochoa-Hein et al., 2021 [43]	Mexico/South America	Hospital-wide/single center	Before–after observational study	January 2019–February 2020 versus Apr–Jul 2020	NA	Overall HAIs	6.2 per 1000 patient days	11.8	0.023
							VAP	10%	54.7%	<0.001
							HAP	26.9%	18.2%	0.025
							BSI	1.3%	20.6%	<0.001
							CAUTI	8.3%	3.5%	0.039
							SSI	25.2%	0.0%	NA
							CDI	15.2%	1.8%	<0.001
							Candidemia	0.0%	8.2%	<0.001
24	Ghali et al., 2021 [24]	Tunisia/Africa	Hospital-wide/single center	Repeated point-prevalence	2019 versus 2020	306 patients versus 296 patients	Overall HAIs	9.5%	15.5%	0.01
25	AlAhdal et al., 2022 [42]	Saudi Arabia/Asia	Hospital-wide/single center	Retrospective observational	January–December 2019 versus January–December 2020	NA	CLABSI	1.2 per 1000 device days	0.5	NA
							CAUTI	0.94 per 1000 device days	0.5	NA
							VAP	1.3 per 1000 device days	0.9	NA
26	Ereth et al., 2021 [57]	USA/North America	Hospital-wide/single center	NA	March–December 2019 versus March–December 2020	NA	NA	6.71 per 1000 patient days	1.03 per 1000 patient days	NA
27	Bentivegna et al., 2021 [45]	Italy/Europe	Medical ward/single center	Retrospective study	2017–2019 versus March–June 2020	NA	CDI	0.066	0.037	NA
28	Choi et al., 2022 [46]	Canada/North America	Hospital-wide/multicenter	Interrupted time series analysis	January 2015–Febr 2020 versus March 2020–June 2021	8,475,872 patient days versus 8,694,620 patient days	CDI	3.4	3.5	0.0896
29	Rosenthal et al., 2022 [39]	Multinational study/Asia and Europe	ICU/multicenter	Pre and post	January–December 2019 versus January–May 2020	7775 patients (pre) versus 1778 patients (pandemic)	CLABSI	2.54 per 1000 line days	4.73 per 1000 line days	0.0006
							CAUTI	1.64 per 1000 catheter days	1.43 per 1000 catheter days	0.690
30	Manea et al., 2021 [48]	Romania/Europe	Hospital-wide/single center	Retrospective cohort	March 2017–February 2018 versus 2020–2021	NA	CDI	6.1 per 1000 adult discharge	5.6 per 1000 discharge	0.600
31	Jabarpour et al., 2021 [23]	Iran/Asia	Hospital-wide/single center	Cross-sectional design	March–July 2019 versus March–July 2020	7454 patients (pre) versus 6135 patients (pandemic era)	Overall HAIs	4.6%	3.7%	0.020
							UTI	0.8%	0.5%	0.040
							BSI	0.8%	0.9%	0.460
							SSI	1.4%	0.9%	0.020
32	Baccolini et al., 2021 [22]	Italy/Europe	ICU/single center	NA	March–April 2019 versus March–April 2020	42 patients (pre) and 62 patients (pandemic era)	Overall HAIs	26.2%	43.6%	NA
33	Whitaker et al., 2022 [44]	USA/North America	Hospital-wide/single center	NA	2019 versus 2020	NA	CAUTI	0.37 per 1000 catheter days	0.23	NA
34	Ramos-Matinez et al., 2020 [58]	Spain/Europe	Hospital-wide/single center	NA	2015–2019 versus March–April 2020	NA	HAI endocarditis	0.119 per 1000 days	0.0194 per 1000 days	<0.001
35	Sipos et al., 2021 [50]	Romania/Europe	Hospital-wide/single center	Retrospective	March–November 2018 & 2019 versus March–November 2020	43,126 patients (pre) versus 25,124 (pandemic era)	CDI	151/43126 (0.36%)	65/25124 (0.26%)	0.0484
								80.8 per 100,000 bed days	70.5 per 100,000 bed days	
36	Lastinger et al., 2022 [40]	USA/North America	Hospital-wide/single center	NA	First, second and third quarters 2019 versus 1st–3rd quarter 2021	1st quarter	CLABSI	0.687	0.998	<0.05
							CAUTI	0.748	0.834	<0.05
							VAE	0.948	1.431	<0.05
							SSI colon surgery	0.866	0.820	>0.05
							SSI abdominal hysterectomy	0.926	0.976	>0.05
							Lab ID CDI	0.628	0.530	<0.05
						2nd quarter	CLABSI	0.697	0.778	<0.05
							CAUTI	0.709	0.706	>0.05
							VAE	0.957	1.209	<0.05
							SSI colon surgery	0.870	0.848	>0.05
							SSI abdominal hysterectomy	0.980	0.988	>0.05
							Lab ID CDI	0.582	0.500	<0.05
						3rd quarter	CLABSI	0.699	1.037	<0.05
							CAUTI	0.705	0.801	<0.05
							VAE	0.999	1.600	<0.05
							SSI colon surgery	0.877	0.796	<0.05
							SSI abdominal hysterectomy	1.087	1.042	>0.05
							Lab ID CDI	0.564	0.482	<0.05
37	Patel et al., 2022 [38]	USA/North America	Hospital-wide/single center	NA	2nd quarter 2019 versus 2nd quarter 2020	NA	CLABSI	0.68	0.87	<0.05

ICU: intensive care unit; CLABSI: central line-associated bloodstream infections; CAUTI: catheter-associated urinary tract infections; CDI: Clostridium difficile infection; SSI: surgical site infections; RVI: respiratory viral infections; HAVI: hospital-acquired viral infections; MDR: multidrug-resistant; HAP: hospital-acquired pneumonia; VAP: ventilator-associated pneumonia; BSI: bloodstream infection; VALRTI: ventilator-associated lower respiratory tract infection; NA: Not available.

**Table 2:** Methodological quality assessment of the studies included in the review.

S/No	Author Name and Year	Selection				Comparability	Outcomes		Quality Score	Quality Scale
		Representatives of Sample	Sample Size	Non-Respondents	Ascertainment of Exposure	Based on Design and Analysis	Assessment of Outcomes	Statistical Test		
1.	Irelli et al., 2020 [26]	*	*	NA	*	*	**	*	7	Good
2.	Alsuhaibani et al., 2022 [28]	*	*	NA	*	*	**	*	7	Good
3.	Sturm et al., 2022 [51]	*	*	NA	*	*	**	*	7	Good
4.	Perez-Granda et al., 2022 [29]	*	*	NA	*	*	**	*	7	Good
5.	Wee et al., 2021 [30]	*	*	NA	*	*	**	*	7	Good
6.	Ochoa-Hein et al., 2021 [47]	*	*	NA	*	*	*	*	6	Fair
7.	Polly et al., 2022 [52]	*	*	NA	*	*	**	*	7	Good
8.	Halverson et al., 2022 [31]	*	*	NA	*	*	**	*	7	Good
9.	Kitt et al., 2022 [53]	*	*	NA	*	*	**	*	7	Good
10.	Advani et al., 2022 [32]	*	*	NA	*	*	**	*	7	Good
11.	Fakih et al., 2022 [33]	*	*	NA	*	*	**	*	7	Good
12.	Teixeira et al., 2022 [54]	*	*	NA	*	*	**	*	7	Good
13.	Ponce-Alonso et al., 2021 [49]	*	*	NA	*	*	**	*	7	Good
14.	Bobbitt et al., 2022 [34]	*	*	NA	*	*	**	*	7	Good
15.	Kong et al., 2021 [36]	*	*	NA	*	*	**	*	7	Good
16.	Tham et al., 2022 [27]	*	*	NA	*	*	**	*	7	Good
17.	Mohammadi et al., 2022 [55]	*	*	NA	*	*	**	*	7	Good
18.	Chen et al., 2021 [25]	*	*	NA	*	*	**	*	7	Good
19.	Losurdo et al., 2020 [56]	*	*	NA	*	*	**	*	7	Good
20.	Geffer et al., 2022 [35]	*	*	NA	*	*	**	*	7	Good
21.	Porto et al., 2022 [37]	*	*	NA	*	*	**	*	7	Good
22.	Samaroo-Campbell et al., 2022 [41]	*	*	NA	*	*	**	*	7	Good
23.	Ochoa-Hein et al., 2021 [43]	*	*	NA	*	*	**	*	7	Good
24.	Ghali et al., 2021 [24]	*	*	NA	*	*	**	*	7	Good
25.	AlAhdal et al., 2022 [42]	*	*	NA	*	*	**	*	7	Good
26.	Ereth et al., 2021 [57]	*	*	NA	*	*	**	*	7	Good
27.	Bentivegna et al., 2021 [45]	*	*	NA	*	*	**	*	7	Good
28.	Choi et al., 2022 [46]	*	*	NA	*	*	*	*	6	Fair
29.	Rosenthal et al., 2022 [39]	*	*	NA	*	*	**	*	7	Good
30.	Manea et al., 2021 [48]	*	*	NA	*	*	*	*	6	Fair
31.	Jabarpour et al., 2021 [23]	*	*	NA	*	*	**	*	7	Good
32.	Baccolini et al., 2021 [22]	*	*	NA	*	*	**	*	7	Good
33.	Whitaker et al., 2022 [44]	*	*	NA	*	*	**	*	7	Good
34.	Ramos-Matinez et al., 2020 [58]	*	*	NA	*	*	*	*	6	Fair
35.	Sipos et al., 2021 [50]	*	*	NA	*	*	**	*	7	Good
36.	Lastinger et al., 2022 [40]	*	*	NA	*	*	**	*	7	Good
37.	Patel et al., 2022 [38]	*	*	NA	*	*	**	*	7	Good

NA: Not applicable; Number of * represents the number of points.

## Data Availability

No new data were created or analyzed in this study. Data sharing is not applicable to this article.
